# Detection of Fastidious Vaginal Bacteria in Women with HIV Infection and Bacterial Vaginosis

**DOI:** 10.1155/2009/236919

**Published:** 2009-11-12

**Authors:** Caroline Mitchell, Carla Moreira, David Fredricks, Kathleen Paul, Angela M. Caliendo, Jaclynn Kurpewski, Jessica Ingersoll, Susan Cu-Uvin

**Affiliations:** ^1^Department of Obstetrics & Gynecology, University of Washington, P.O. Box 356460, Seattle, WA 98195, USA; ^2^Department of Obstetrics & Gynecology, Brown University, Providence, RI 02912, USA; ^3^Fred Hutchinson Cancer Research Center, Seattle, WA 98109, USA; ^4^Department of Pathology and Laboratory Medicine, Emory University, Atlanta, GA 30322, USA; ^5^Emory Center for AIDS Research, Emory University, Atlanta, GA 30322, USA

## Abstract

*Background*. Fastidious bacteria have been associated with bacterial vaginosis (BV) using PCR methods. We assessed the prevalence of these bacteria in HIV-1 infected women and their relationship with vaginal pH and shedding of HIV-1 RNA. *Methods*. 64 cervicovaginal lavage (CVL) samples were collected from 51 women. Vaginal microbiota were characterized using 8 bacterium-specific quantitative PCR assays. *Results*. Women with the fastidious bacteria Bacterial Vaginosis Associated Bacterium (BVAB) 1, 2, and 3 showed a trend to increased HIV-1 shedding (OR 2.59–3.07, *P* = .14–.17). Absence of *Lactobacillus crispatus* (*P* < .005) and presence of BVAB2 (*P* < .001) were associated with elevated vaginal pH. BVAB1, 2, and 3 were highly specific indicators of BV in HIV-infected women, with specificities of 89%–93%. *Conclusions*. Fastidious bacteria (BVAB 1, 2, and 3) remain specific indicators of BV in HIV-infected women, and BVAB2 may contribute to the elevated vaginal pH that is a hallmark of this syndrome.

## 1. Background

Bacterial vaginosis (BV) is a vaginal infection characterized by loss of the normal protective lactobacilli and overgrowth of diverse anaerobes [1, 2]. This infection is one of the leading causes of vaginal discharge [3] and is more prevalent in HIV-1-infected women compared to uninfected women [4]. The microbiology of BV is heterogeneous, and culture-based description of the vaginal microbiota identifies far fewer organisms than broad range molecular methods [5, 6]. Several fastidious bacteria in the *Clostridiales* order have recently been associated with BV using PCR methods [7] and appear to be highly specific markers of BV [8].

BV has been associated with increased genital HIV shedding [9], having high concentrations of *Gardnerella vaginalis* and *Mycoplasma hominis* [10], but the mechanism accounting for this association is poorly understood. In HIV-1-infected women, polymerase chain reaction (PCR) methods have shown that high concentrations of *Gardnerella vaginalis* and *Mycoplasma hominis* are sensitive indicators for the diagnosis of BV [11]. It is possible that some of the proinflammatory vaginal cytokines seen in subjects with BV [12, 13] increase production of the virus or stimulate epithelial turnover and microtears in the vaginal wall that facilitate viral shedding. A second hypothesis is that loss of hydrogen peroxide-producing lactobacilli and subsequent increase in vaginal pH results in loss of viral inhibition [14]. Lactobacilli and *G. vaginalis* have been associated with changes in vaginal pH [15], but little is known about the effect of the fastidious organisms present in BV.

We hypothesize that fermentation products from key vaginal anaerobes may alter the vaginal pH and facilitate production and shedding of HIV. We sought to determine the prevalence of several fastidious vaginal bacteria in HIV-infected women and to assess the impact of these bacterial species on (1) vaginal pH and (2) shedding of HIV-1 RNA in women with and without BV.

## 2. Methods

### 2.1. Sample Collection and Characterization

This was a secondary analysis of samples collected as part of a prospective cohort study of nonpregnant HIV-1-infected women in Providence, Rhode Island, evaluating the effect of antiretroviral therapy (ART) on HIV-1 RNA shedding in the female genital tract [16]. In the parent study, women were seen at baseline, 2 weeks, one month, and every 6 months for 36 months. All patients gave informed consent to participate in the study; the study protocol was approved by the Institutional Review Board of The Miriam Hospital, Providence, RI. At each visit clinical data and exam findings were recorded, and blood and cervicovaginal lavage (CVL) samples were collected. Women were asked not to douche, have sex, or use any intravaginal product for the 48 hours prior to the visit. The CVL was collected by infusing 10 cc of normal saline into the vagina with a syringe and then aspirating back the fluid. For this subanalysis samples were randomly selected to represent approximately equal numbers of samples from women with and without BV, and from women who were on antiretroviral therapy and those who were not. Not all selected samples were available for analysis, and so the final numbers in each category are not exactly equal (e.g., 34 samples from subjects with BV and 30 samples from subjects without BV). Some women contributed samples from more than one visit, but each sample was analyzed as an individual measurement, and GEE equations were used in the statistical analysis to account for this.

Bacterial vaginosis was diagnosed using Amsel's clinical criteria [17]: clinicians assessed vaginal discharge, pH, and presence of clue cells and fishy odor with addition of KOH and patients with at least 3 of 4 criteria were considered to have BV. Trichomonas and Candida vaginitis were also diagnosed clinically using wet mount. Previous studies in this population had shown that the prevalence of syphilis, gonorrhea, and chlamydia were quite low; so testing was only done annually [18]. HIV-1 RNA was quantified using the nucleic acid sequence-based assay (Nuclisens, bioMerieux, Durham, NC), with a sensitivity of 400 copies/mL. Cells in the vaginal fluid were classified by an experienced Medical Technologist as white blood cells, red blood cells, or nucleated cells (primarily vaginal epithelial cells). Manual cell counts were performed on a hemocytometer, counting cells in five 1 mm squares and averaging the results.

### 2.2. Sample Processing and Testing

The CVL sample was thawed and then centrifuged for 10 minutes at 14000 × g, and the supernatant removed. The remaining pellet underwent DNA extraction with the MoBio UltraClean Soil DNA Isolation Kit (MoBio,Calsbad, CA) following the manufacturer's instructions. A clean swab was taken through the DNA extraction process as a negative extraction control. All extracted DNA samples and the extraction control were tested in a quantitative PCR using primers targeting the human 18S rRNA gene to validate that successful DNA extraction occurred. An internal amplification control PCR using exogenous DNA from a jellyfish gene was used to test for presence of PCR inhibitors [19]. The presence of inhibition is defined as an internal amplification control qPCR threshold cycle value that was 2 cycles higher than that of the no-template control.

Patient samples were then subjected to eight separate taxon-directed 16S rRNA gene quantitative PCR assays for the detection and quantification of individual bacteria: *Lactobacillus* genus, *Lactobacillus crispatus*, *Gardnerella vaginalis*, *Leptotrichia/Sneathia*, *Megasphaera*, and Bacterial Vaginosis-Associated Bacterium (BVAB) 1, BVAB2, and BVAB3. One assay detects two bacterial species (*Leptotrichia* and *Sneathia*) which are closely related. The BVAB are related to bacteria in the *Clostridiales* order and have been found to be highly specific for BV [7]. Each assay has previously been validated and proven to be sensitive (to a level of 1–10 DNA copies/reaction, or 150–1500 copies/mL) and specific (does not detect other bacteria at a concentration of 10^6^ copies/rxn) [20]. The assays use a TaqMan format and were run on an ABI 7500 Thermocycler (Applied Biosystems, Foster City, CA). Plasmids containing bacterial 16S rRNA genes were used to generate standard curves for quantification. The standards were generated by cloning bacterial 16S rRNA genes into *E. coli* and then purifying plasmids. The plasmids were quantified using a fluorimeter and the Quant-iT Pico Green assay kit (Invitrogen, Carlsbad, CA) to determine the number rRNA gene copies per microliter.

### 2.3. Statistical Analysis

All analyses were carried out using Stata v9.2 (StataCorp, College Station, Texas). Demographic variables were compared between groups using the Mann-Whitney U-test for continuous variables and Independent samples *t *-test for categorical variables. Log-transformed concentrations of bacteria were compared between women with and without BV using a *t*-test. The relationships between presence and absence of bacteria and detection of HIV and sensitivity and specificity for diagnosis of BV were modeled using logistic regression and generalized estimating equations to account for repeat measures. Linear regression was used to model the relationship between bacterial species and continuous variables such as quantity of nucleated cells. For all regression analyses, the method of generalized estimating equations (GEEs) with robust standard errors was used to account for residual correlation due to the fact that some observations were repeated measures on the same women over time. Our power calculation was based on the 70 samples initially selected, and we estimated an 80% power to detect a twofold increase in the rate of HIV-1 shedding in women with detectable BV-associated bacteria.

## 3. Results

Sixty-four CVL samples from 51 women were analyzed. One woman contributed three samples, and eleven women contributed two samples. When comparing only the first sample from each subject, 24 women with BV and 27 women without BV were similar in terms of age, race, time since HIV-1 diagnosis, antiretroviral treatment status, CD4 count, and plasma viral load (Table 1). Initially 65 samples were processed, but one sample did not contain adequate material for analysis. All other samples had detectable DNA extracted, and no evidence of PCR inhibition. 

Sixty (94%) of the 64 samples had any lactobacilli detected by genus specific PCR, but only 23 (37%) had *Lactobacillus crispatus* detected. *Gardnerella vaginalis* was detected in 84% of samples, *Megasphaera* in 31%, *Leptotrichia/Sneathia* in 59%, BVAB1 in 19%, BVAB2 in 41%, and BVAB3 in 27%. As in previous studies, not all bacteria were detected in all women with BV, and the novel bacteria BVAB1, BVAB2, and BVAB3 were detected in some women without BV though at a lower prevalence. Furthermore, the concentrations of *Leptotrichia/Sneathia*, *Megasphaera*, BVAB1, BVAB2, and BVAB3 were significantly lower in women without BV compared to women with BV (Table 1). 

After controlling for plasma viral load there were no significant associations between HIV-1 RNA in the genital tract and detection of any individual bacteria; however there were trends toward increased HIV shedding in subjects with BVAB 1, 2, and 3 (OR 2.59–3.07, *p* = .14–.17) irrespective of BV diagnosis (Table 2). The largest odds ratio, while still not significant, was for an association of vaginal HIV-1 RNA with detection of *Gardnerella vaginalis* (OR 17.2, 95% CI 0.48,619). There were no significant differences in the quantities of individual bacteria between women with CD4 counts >500 cells/mL, 200–500 cells/mL, and <200 cells/mL (data not shown). 

Novel bacteria in the *Clostridiales* order-designated BVAB1, BVAB2, and BVAB3 remain highly specific indicators of BV in HIV infected women, with specificities of 89%–93% (Table 3). However, BVAB2 is a more sensitive indicator of BV (63%) and when detection of either BVAB2 or *Megasphaera* is used as a diagnostic criterion sensitivity improves to 71%. As has been established in other studies [21], high concentrations of *Gardnerella vaginalis* are also sensitive for the diagnosis of BV and more specific than the mere presence of the organism. 

Higher concentrations of *Lactobacillus crispatus* were associated with a lower vaginal pH. Higher concentrations of *Gardnerella vaginalis*, *Leptotrichia/Sneathia*, *Megasphaera*, BVAB1, BVAB2, and BVAB3 were all associated with an increase in vaginal pH (Table 4). In a multivariate model, quantities of *Lactobacillus crispatus, Gardnerella vaginalis*, and BVAB2 are most independently predictive of vaginal pH (*p* = .004, *p* = .047, and *p* < .001, resp.). The presence of any *Lactobacillus* species was associated with a significant decrease in number of nucleated cells (primarily epithelial cells) in CVL (*p* = .001), while *Gardnerella vaginalis *was associated with a significant increase (*p* = .015) (Table 4).

## 4. Discussion

Several fastidious vaginal bacterial species are associated with BV in HIV-1-infected women. Detection of the fastidious bacterium BVAB2 in combination with *Megasphaera* species provides a reasonably sensitive marker for the diagnosis of BV, consistent with findings in HIV-1-uninfected women [8]. 

The impact of bacterial vaginosis on vaginal health may be mediated in several ways. Proinflammatory cytokine levels increase in women with BV [12] which may cause epithelial damage or recruit immune cells capable of HIV replication. However, the association between *Gardnerella vaginalis* and the presence of more nucleated cells in the vagina suggests that the BV-associated biofilm, of which *G. vaginalis *is a significant component, may cause direct effects on the epithelial surface and may be a mechanism to facilitate entry or shedding of HIV. 

It is not surprising that the absence of *L. crispatus *is associated with higher vaginal pH, but the significant effect of the BVABs on vaginal pH is a new finding. Given the strongly significant *P* values for this finding in spite of the small sample size, it is likely that this is a true biologic phenomenon that may be related to fermentation products produced by these fastidious bacteria as well as *G. vaginalis*, *Megasphaera*, *Leptotrichia, * and *Sneathia spp *[22]. Lower pH has been shown to inactivate HIV-1 [23]; thus any organism that increases the normally low vaginal pH may facilitate replication and proliferation of HIV-1. 

We did not see a relationship between quantities of any individual bacteria and vaginal shedding of HIV, which is in contrast to a previous study that looked at the relationship between concentrations of *Lactobacillus* species, *Gardnerella vaginalis, * and *Mycoplasma hominis* [10]. That study had significantly more women, all of whom had a log higher detectable plasma viral load than the women in our study and none of whom were on highly active ART, thus making it significantly more likely that they would have detectable vaginal HIV shedding and power to detect associations. As our understanding of the bacterial diversity of BV grows, it seems less likely that one individual bacterium will be associated with all adverse outcomes in all women. Rather, we suspect that the loss of protective lactobacilli with a resulting rise in pH and production of multiple fermentation products by anaerobes work together to produce negative effects such as increased shedding of HIV-1 [24]. 

There are several limitations to this study. The small study population and heterogeneity of bacterial communities in individual women result in limited power to detect relationships between individual bacteria and HIV shedding. Although individual samples were selected randomly, not all samples were available and thus this analysis does not represent a true random sampling of the parent cohort. The lack of an HIV-1-uninfected control group means that these results may not be generalizable to women without HIV infection. However, the prevalence and quantities of bacteria described in this population are similar to those reported in an HIV-1 negative population, as is the heterogeneity between individuals [8]. One difference in this cohort is that the prevalence and concentration of *Lactobacillus crispatus* was the same between women with and without BV. This may be an artifact of sample selection but suggests that this cohort may have unique features. Additionally, BV was diagnosed by clinical criteria, while Gram-stain criteria are frequently used as a gold standard in research. In this type of analysis we feel that the clinical diagnostic criteria are quite relevant. Since the Gram stain criteria give a higher score to women with more *G. vaginalis* morphotypes [25], it would not be surprising to find that women with BV diagnosed by Gram stain have higher concentrations of *G. vaginalis* using PCR. Thus, comparing bacterial qPCR results to clinical criteria is a more independent comparison. The contribution of other genital tract infections to these results is difficult to assess. Coinfection with gonorrhea, Chlamydia, or syphilis was previously seen to be very low in this cohort [18] but was not measured at each visit. The use of wet mount to diagnose yeast or Trichomoniasis has low sensitivity [26, 27] and may have misdiagnosed some women. 

Several bacteria found in HIV-1-infected women with BV may impact HIV shedding through their impact on promoting turnover of nucleated vaginal epithelial cells (*Gardnerella vaginalis*) or by increasing vaginal pH (BVAB2). There was a trend suggesting that BVAB1, BVAB2, and BVAB3 may be associated with increased shedding of HIV-1, but this hypothesis will need to be confirmed or refuted in larger studies.

## Figures and Tables

**Table 1:** Characteristics of the study population (*N* = 51^§^).

	BV + (*N* = 24)	BV − (*N* = 27)	*P* value
Age	35.7 ± 8.6	38.2 ± 6.5	.23
Race			
White	11 (46)	9 (33)	.83
African American	8 (33)	11 (41)	
Hispanic	5 (21)	7 (26)	
(*2 missing*)			
Time since HIV diagnosis	7.4 ± 4.8	6.8 ± 4.0	.59
On ART	9 (38)	13 (46)	.42
CD4 Count (median, IQR) cells/mL	393 (246–582)	437 (209–619)	.74
Plasma viral load (median, IQR) copies/mL	3250 (800–44,500)	4800 (320–41,000)	>.99
Vaginal fluid cells (cells/cc)			
Nucleated cells	961 ± 1296	752 ± 1034	.53
RBCs	39 ± 41	676 ± 2458	.21
Monocytes	2.9 ± 5.4	0.2 ± 0.6	.012
Lymphocytes	0.8 ± 1.6	0.5 ± 1.3	.50
Eosinophils	1.2 ± 1.8	0.3 ± 1.0	.04
Log-transformed bacterial 16S rDNA copies/mL (mean ± SD)			
*Lactobacillus* genus	6.93 ± 1.74	6.71 ± 2.08	.69
*Lactobacillus crispatus *	2.36 ± 3.05	2.43 ± 3.30	.94
*Gardnerella vaginalis *	7.21 ± 1.82	3.70 ± 3.04	<.001
*Megasphaera *	3.56 ± 3.66	0.56 ± 1.71	<.001
*Leptotrichia/Sneathia *	5.82 ± 3.59	2.12 ± 3.23	<.001
BVAB1	2.06 ± 3.51	0.50 ± 1.85	.045
BVAB2	4.22 ± 3.61	0.48 ± 1.50	<.001
BVAB3	2.38 ± 3.15	0.50 ± 1.50	.007

^§^For individuals who contributed more than one sample, only data from the first visit are included in this table.

*Tests are chi-square for categorical variables, and *t*-test or Mann-Whitney for continuous variables.

Data presented as mean ± SD for continuous variables or *n*(%) for categorical variables unless otherwise noted.

**Table 2:** Odds ratio for having detectable HIV-1 in cervicovaginal lavage when individual bacteria are detected by PCR.

Bacterial species	*N* (%)^a^	OR	95% CI	*P* value
(any versus none)				
*Lactobacillus* spp.	60 (94%)	N/A^b^	—	—
*Lactobacillus crispatus*	23 (37%)	3.17	0.85, 11.8	.09
*Gardnerella vaginalis*	54 (84%)	17.2	0.48, 619	.12
*Leptotrichia/Sneathia*	38 (59%)	0.61	0.17, 2.13	.44
*Megasphaera* spp.	20 (31%)	0.91	0.26, 3.17	.88
BVAB1	12 (19%)	3.07	0.68, 14.0	.15
BVAB2	26 (41%)	2.59	0.74, 9.0	.14
BVAB3	17 (27%)	3.03	0.63, 14.7	.17

^a^Number of women with bacteria detected by qPCR. ^b^Cannot be computed, because only 4 women had no lactobacilli, and all had detectable cervicovaginal HIV shedding (OR would be infinity).

**Table 3:** Sensitivity and specificity of detection of individual and combinations of bacteria for diagnosis of bacterial vaginosis compared to Amsel's clinical criteria.

	Sensitivity	Specificity	OR (95% CI)	*P* value
Any *Lactobacillus* spp.	0%	100%	1.8 (0.15, 20.8)	.65
Any *Lactobacillus crispatus *	42%	63%	1.2 (0.39, 3.75)	.74
Any *Gardnerella vaginalis *	95%	32%	10.9 (1.3, 94)	.03
Any *Leptotrichia/Sneathia *	75%	68%	6.3 (1.8, 21)	.003
Any *Megasphaera* spp	50%	89%	8.3 (2.0, 35)	.004
Any BVAB1	29%	93%	5.4 (0.99, 29)	.051
Any BVAB2	63%	89%	13.9 (3.2, 60)	<.001
Any BVAB3	38%	89%	5.0 (1.2, 21)	.02
Any *Megasphaera* spp. OR BVAB2	71%	86%	14.6 (3.7, 58)	<.001
High *Gardnerella vaginalis* (>median)	75%	82%	13.8 (3.6, 53)	<.001

**Table 4:** Vaginal effects of bacteria: univariate correlation (using linear regression) between presence of bacterial species and log-transformed quantity of nucleated cells in the CVL and between concentration of bacterial species and pH of the vaginal secretions.

	Nucleated cells			pH		
	(model with presence/absence bacterial species)			(model with concentration of bacterial species)		
Bacterial species	Regression coefficient	95% CI	*P* value	Regression coefficient	95% CI	*P* value
*Lactobacillus* spp	−0.600	−0.958, −0.242	.001	0.019	−0.092, 0.129	.74
*Lactobacillus crispatus*	0.136	−0.185, 0.456	.41	−0.127	−0.18, −0.073	<.001
*Gardnerella vaginalis*	0.573	0.110, 1.035	.015	0.156	0.098, 0.214	<.001
*Leptotrichia/Sneathia*	0.226	−0.083, 0.535	.15	0.123	0.078, 0.169	<.001
*Megasphaera* spp.	0.130	−0.196, 0.456	.43	0.108	0.062, 0.154	<.001
BVAB1	0.012	−0.376, 0.400	.95	0.062	0.007, 0.118	.027
BVAB2	0.042	−0.267, 0.351	.79	0.160	0.11, 0.204	<.001
BVAB3	−0.021	−0.364, 0.321	.90	0.113	0.058, 0.168	<.001
